# Generation of V α13/β21^+^T cell specific target CML cells by TCR gene transfer

**DOI:** 10.18632/oncotarget.12441

**Published:** 2016-10-04

**Authors:** Xianfeng Zha, Ling Xu, Shaohua Chen, Lijian Yang, Yikai Zhang, Yuhong Lu, Zhi Yu, Bo Li, Xiuli Wu, Wenjie Zheng, Yangqiu Li

**Affiliations:** ^1^ Department of Clinical Laboratory, First Affiliated Hospital, Jinan University, Guangzhou, China; ^2^ Institute of Hematology, Jinan University, Guangzhou, China; ^3^ Department of Chemistry, Jinan University, Guangzhou, China; ^4^ Department of Hematology, First Affiliated Hospital, Jinan University, Guangzhou, China; ^5^ Key Laboratory for Regenerative Medicine of Ministry of Education, Jinan University, Guangzhou, China

**Keywords:** chronic myeloid leukemia, T cell receptor, gene transfer, immunotherapy

## Abstract

Adoptive immunotherapy with antigen-specific T cells can be effective for treating melanoma and chronic myeloid leukemia (CML). However, to obtain sufficient antigen-specific T cells for treatment, the T cells have to be cultured for several weeks *in vitro*, but *in vitro* T cell expansion is difficult to control. Alternatively, the transfer of T cell receptors (TCRs) with defined antigen specificity into recipient T cells may be a simple solution for generating antigen-specific T cells. The objective of this study was to identify CML-associated, antigen-specific TCR genes and generate CML-associated, antigen-specific T cells with T cell receptor (TCR) gene transfer. Our previous study has screened an oligoclonal Vβ21 with a different oligoclonal Vα partner in peripheral blood mononuclear cells (PBMCs) derived from patients with CML. In this study, oligoclonally expanded TCR α genes, which pair with TCR Vβ21, were cloned into the pIRES eukaryotic expression vector (TCR Vα-IRES-Vβ21). Next, two recombinant plasmids, TCR Vα13-IRES-Vβ21 and TCR Vα18-IRES-Vβ21, were successfully transferred into T cells, and the TCR gene-modified T cells acquired CML-specific cytotoxicity with the best cytotoxic effects for HLA-A11^+^ K562 cells observed for the TCR Vα13/Vβ21 gene redirected T cells. In summary, our data confirmed TCRVα13/Vβ21 as a CML-associated, antigen-specific TCR. This study provided new evidence that genetically engineered antigen-specific TCR may become a druggable approach for gene therapy of CML.

## INTRODUCTION

Chronic myelogenous leukemia (CML) is a common hematological malignancy in adult. The typical genetic alteration is the Philadelphia chromosome resulting from,t(9;22) (q34;q11), which forms a *bcr-abl* fusion gene encoding BCR-ABL fusion proteins with unusual tyrosine kinase activity [1]. Therefore, tyrosine kinase inhibitors (TKIs) such as imatinib were developed as ATP competitive inhibitors of the bcr-abl tyrosine kinase fusion protein for CML therapy [2]. Compared with previous standard therapy, treatment with imatinib have improved significantly the outcome of the patients with CML. However, approximately 30% of patients interrupt imatinib therapy because of suboptimal response or intolerance, in the case, the second-generation TKIs are the choice for the patients [3, 4]. It is well known, allogenic hematopoietic stem cell transplantation (allo-HSCT) is currently the only curative therapeutic approach for CML. However, the application of such procedure is suitable only for approximately 30% of CML patients due to the limitation of the availability of matched donors and the toxicity in older patients [5, 6]. Adoptive T cell immunotherapy is an effective alternative for treating CML patients, particularly patients with relapsed CML after HSCT. Donor lymphocyte infusion (DLI) has improved the outcome of relapsed CML patients after allo-HSCT, which has replaced IFN-α as the preferred treatment for relapsed CML after HSCT [7, 8]. Infused donor-derived cytotoxic T lymphocytes (CTLs) recognize leukemia associated antigens expressed by CML cells, resulting in CTL-mediated leukemia cell death. Unfortunately, a part of CTL-recognized also allo-antigens which are expressed in host normal tissues, which can lead to graft-versus-host disease (GVHD). Hence, the ideal strategy for adoptive T cell immunotherapy is to infuse leukemic antigen-specific cytotoxic T lymphocytes (CTLs). However, application of this mode of leukemic antigen-specific T cell adoptive transfer is often limiting because the isolation and *in vitro* expansion of leukemic antigen-specific T cells is labor-intensive and time-consuming [9]. Fortunately, a recently developed T cell receptor (TCR)-mediated gene therapy may facilitate overcoming this limitation.

TCRs include α, β, γ and δ chains, most circulating mature T cells use the α/β heterodimeric TCR for specific recognition of antigenic peptides presenting by major histocompatibility complex (MHC) molecules from antigen presenting cells. The specific TCRs could be identified by characterizing the rearrangement of TCRα and TCR β genes. Transfer of antigen-specific TCR genes into recipient T cells using transgenic method will lead to the transfer of leukemic-specific T cell immunity. Therefore, specific TCR gene transfer is an attractive strategy for the fast *in vitro* generation of sufficient numbers of antigen-specific T cells [9]. To date, the successful transfer of TCR genes specific for virus-specific and tumor-associated antigens, such as EBV and MART-1 and Wilms' tumor antigen 1 (WT1), has been shown to have specific cytotoxicity for EBV+ lymphoma, leukemia and melanoma [10–13]. However, little is known about the TCR genes specific for CML-associated antigens.

Previously, we identified specific TCR gene sequences related with a CML-associated antigen, which was submitted to GenBank (the accession number: GU997647). In this study, we developed recombinant constructs containing HLA-A11-restricted TCRα13 and TCRβ21 genes specific for CML-associated antigens, and showed that the TCR gene-modified T cells had the specific cytotoxicity toward the HLA-A11+ K562 cell line. The results may indicate that it is viable to prepare leukemic antigen specific T cells from polyclonally expanded T cells when the MHC -restricted TCR genes are identified.

## RESULTS

### Cloning of TCRs from CML patient and construction of TCR bicistronic eukaryotic expression plasmid

**Figure 1:**
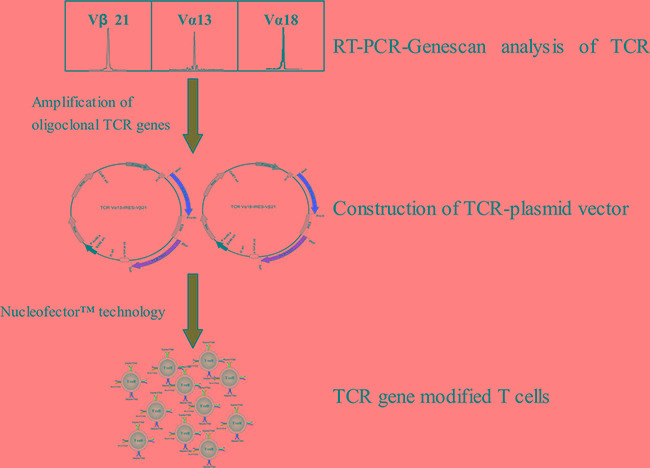
The schematic diagram of generation of CML associated antigen-specific T cells by TCR gene transfer

**Figure 2:**
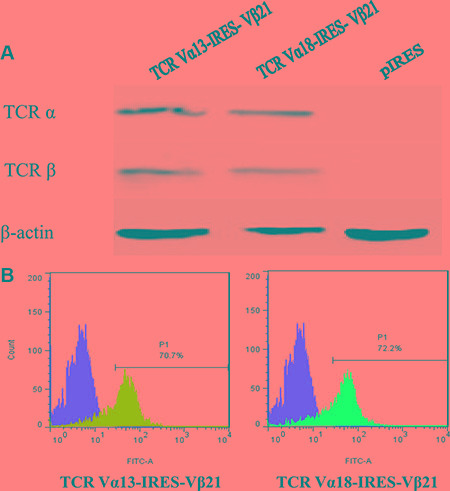
Expression of TCR α13/β21 and α18/β21 on HEK 293 cells and Jurkat T cells transduced with TCR Vα13-IRES-Vβ21 and Vα18-IRES-Vβ21 (**A**) Western blot analysis of TCR α13, α18 and β21 on HEK 293 cells transdued with TCR Vα13-IRES- Vβ21 , Vα18-IRES-Vβ21 and pIRES. (**B**) Transduced Jurkat T cells were stained with mouse anti-human TCR Vβ21.3 monoclonal antibody and analyzed by flow cytometry.

In our previous study, oligoclonally expanded TCR α13, α18 and β21 subfamily T cells were identified in the PB of patients with CML [14]. In this study, full length TCR α13, α18 and β21-chain genes were amplified by PCR, and the TCR α13 and α18 genes, which pair with TCR β21, were then cloned into the pIRES eukaryotic expression vector to construct two bicistronic recombinant plasmids, TCR α13-IRES-β21 and TCR α18-IRES-β21 (Figure 1). Subsequently, their sequence were verified by restriction enzyme digestion and sequencing (data not shown). To confirm expression of the TCR α13 and TCRβ21 chains, the TCR-encoding expression plasmids were first transfected into HEK 293 cells using lipofection technology. After transfection, the TCR α13/β21 and α18/β21 proteins could be detected by Western blotting (Figure 2A). Additionally, the TCR β21 protein could be determined on Jurkat T cells labeled with a mouse anti-human TCR β21-FITC monoclonal antibody by FCM (70.2% and 72.2%) (Figure 2B). These results indicate that the recombinant plasmid could be successfully expressed in eukaryotic cells.

### TCR α/β gene-modified CD3^+^T cells

**Figure 3:**
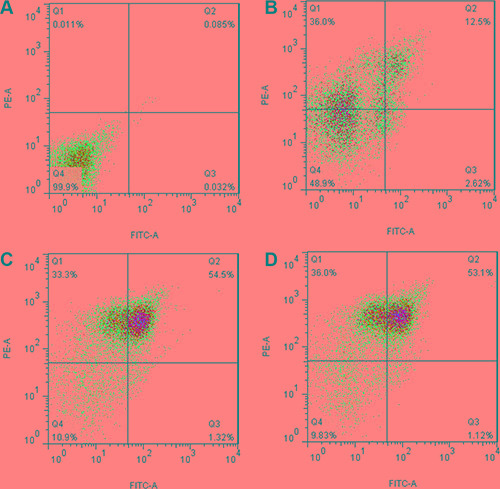
Expression of TCR α13/β21 and α18/β21 on CD3^+^ T cells transduced with pIRES, TCR Vα13-IRES-Vβ21 and TCR Vα18-IRES-Vβ21 Transduced cells was were stained with mouse anti-human TCR Vβ21.3 monoclonal antibody and analyzed by flow cytometry. (**A**) Isotype Control; (**B**) pIRES; (**C**) TCR Vα13-IRES- Vβ21; (**D**) TCR Vα18-IRES-Vβ21.

CD3^+^T cells were transfected with the TCR α13-IRES-β21 and TCR α18-IRES-β21 recombinant plasmids using Nucleofector™ technology. Twenty-four hours after transfection, the efficiencies of gene transfer were determined by FCM which detected the TCR β21 on the CD3^+^T cells (Figure 3). The TCR transfer efficiency of the TCR α13-IRES-β21 and TCR α18-IRES-β21 recombinant plasmids was 43.4 ± 10.3% and 39.6 ± 5.0%, respectively. These data indicated that the two recombinant plasmids could express the inserted proteins in normal T cells, and the expression efficiency could satisfy subsequent toxicity tests.

### Specificity and functionality of TCR-transduced T cells

We used multiple approaches to ensure that the TCR gene-modified CD3^+^T cells were specific and active against antigen-expressing targets.

To test the TCR gene specificity, target cells must express corresponding MHC class-I molecules. K562 cells do not express MHC class-I molecules [15]; thus, to overcome this limitation, K562 cells were modified to express HLA-A*11 molecules [16]. In addition, negative control HEK293 cells were also modified to express HLA-A*11 molecules.

**Figure 4:**
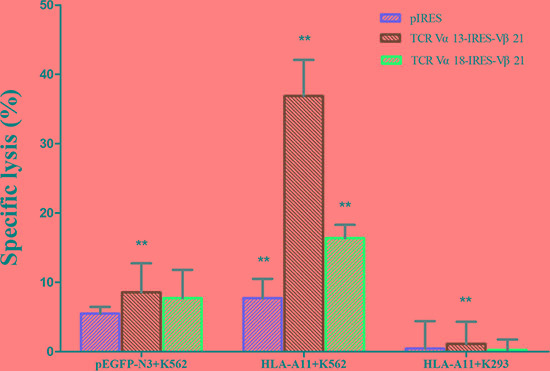
Specific cytotoxicity of TCR gene-transduced CD3^+^T cells against HLA-A11^+^ K562 cells as determined by calcein-AM release assay Three days after transduction, CD3^+^T cells transduced with pIRES, TCR Vα13-IRES-Vβ21, and TCR Vα18-IRES-Vβ21 were co-cultured with pEGFP-N3^+^K562, HLA-A11^+^ K562 and HLA-A11^+^ K293 cells at a 40:1 ratio for 4 h. Then, the calcein-AM level in the supernatant was determined. The spontaneous release of calcein-AM from both target and effector cells was subtracted from the measured values, and the final results are expressed as the percentage of specific cytotoxicity. ** , < 0.05. Error bars, S.D.

Having verified that the exogenous TCRs were expressed equally in T cells and the HLA-A*11^+^ K562 target cell line, we evaluated the ability of the TCR-modified T cells to lyse their target cells using the calcein-AM release assay. The killing efficiency was tested following the co-cultivation of effector cells (TCR α13-IRES-β21, TCR α18-IRES-β21 or pIRES-transduced T cells) with target cells (pEGFP-N3^+^ K562, HLA-A*11^+^ K562, and HLA-A*11^+^ K293 cells). The specific killing efficiency of TCR α13/β21-transduced T cells toward HLA-A*11^+^ K562 cells was significantly higher than that for pEGFP-N3^+^ K562 (*p* = 0.002) and HLA-A*11^+^ K293 (*p* = 0.001) cells. The specific killing efficiency of TCR α18/β21-transduced T cells for the three target cells demonstrated differences, but these were not statistically significant. The specific killing efficiency of TCR α13/β21-transduced T cells toward HLA-A*11^+^ K562 cells was significantly higher than that of TCR α18/β21-transduced (*p* = 0.005) and pIRES-transduced (*p* = 0.003) T cells. In co-cultivation experiments, we found that TCR α13/β21-transduced T cells had the highest killing efficiency for the HLA-A*11^+^ K562 cell line (Figure 4), indicating that the TCR α13/β21-modified T cells were specifically directed against CML cells.

### Expression of granzyme B and perforin in TCR-transduced T cells

**Figure 5:**
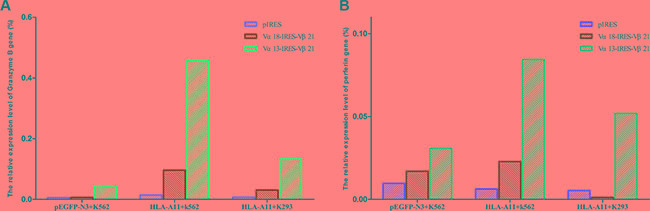
mRNA expression of granzyme B (A) and perforin (B) Three days after transduction, CD3^+^T cells transduced with pIRES, TCR Vα13-IRES-Vβ21 and Vα18-IRES-Vβ21 were co-cultured with pEGFP-N3^+^K562, HLA-A11^+^K562 and HLA-A11^+^K293 cells at a 40:1 ratio for 6 hours. Then, total RNA was extracted and the expression of granzyme B and perforin were detected by RT-PCR.

Granzyme B and perforin are expressed by CTLs and play an important role in their cytotoxic activity. Perforin is required for cytolysis, and granzymes efficiently induce target cell death; thus, the granzyme B and perforin expression level in TCR-transduced T cells can reflect cytotoxicity strength. We found that the granzyme B and perforin expression level in TCR α13/β21-transduced T cells in response to HLA-A*11^+^ K562 cells was 4.8- and 3.6-fold greater, respectively, than that in TCR α18/β21-transduced T cells, and the levels were 31.6- and 13.5-fold greater than that in empty plasmid transfected T cells. In addition, the expression level of granzyme B and perforin in TCR α13/β21-transduced T cells in response to HLA-A11^+^K293 and pEGFP-N3^+^K562 cells was also greater than that in TCR α18/β21-transduced T cells and empty plasmid-transfected T cells (Figure 5).

### IFN-γ secretion in TCR-transduced T cells

**Figure 6:**
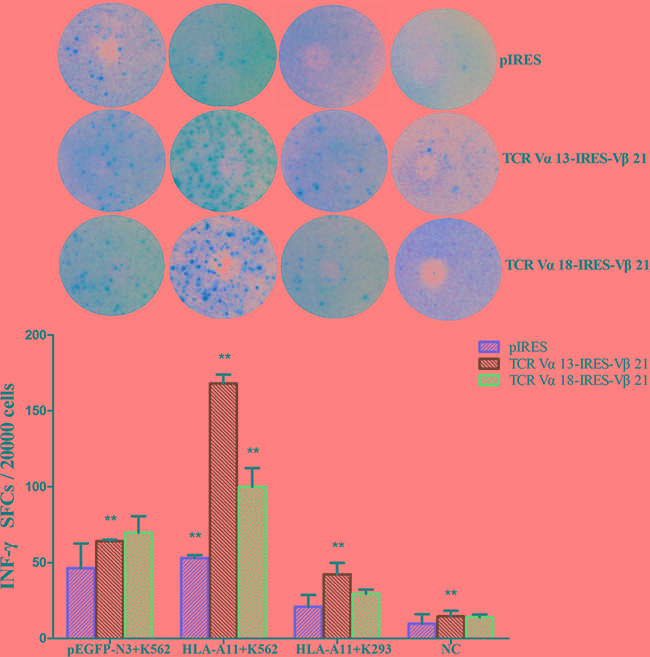
INF-γ levels in TCR Vα13-IRES-Vβ21 and TCR Vα18-IRES-Vβ21 transfected CD3^+^ T cells Three days after transduction, CD3^+^T cells transduced with pIRES, TCR Vα13-IRES-Vβ21 and Vα18-IRES-Vβ21 were co-cultured with pEGFP-N3^+^K562, HLA-A11^+^K562 , and HLA-A11^+^K293 cells plus a negative control at a 40:1 ratio for 6 hours. Then, The INF-γ was analyzed by ELISPOT array. *The Mann-Whitney test for two independent samples was used to determine differences between various groups. Statistical significance was defined as *P* < 0.05. ** , < 0.05. Error bars, S.D.

IFN-γ secretion is a symbol of T cell activation. Thus, TCR-transduced T cells were tested for IFN-γ release in response to each target cell using specific ELIspot assays. We found that the IFN-γ production of TCR α13/β21-transduced T cells (168 ± 5.8 SFC/2 × 10^4^ cells) in response to HLA-A*11^+^ K562 cells was greater than that of empty plasmid-transfected T cells (58 ± 1.8 SFC/2 × 10^4^ cells) (*p <* 0.05), and it was also greater than its IFN-γ production in response to the pEGFP-N3^+^ K562 K562 (64 ± 1.09 SFC/2 × 10^4^ cells) and HLA-A*11^+^ K293 (42 ± 7.5 SFC/2 × 10^4^ cells) cell lines. A similar result was observed for TCR α18/β21-transduced T cells. However, the IFN-γ production of TCR α13/β21-transduced T cells in response to the HLA-A*11^+^ K562 line was greater than that for TCR α18/β21-transduced T cells (Figure 6). These results suggest that both TCR α13/β21- and α18/β21-transduced T cells specifically respond to CML cells, and TCR α13/β21-transduced T cells have the best response.

## DISCUSSION

Increasing studies have demonstrated that adoptive transfer of TCR transgenic T cells is a promising strategy for treating patients suffering from malignancies [17–19]. For clinical application of transgenic TCR T cells, obtaining tumor-associated, antigen-specific TCR from tumor-specific CTLs is a prerequisite. In CML, the p210 protein encoded by the BCL-ABL fusion gene has a unique advantage for obtaining antigen-specific TCR genes [20, 21]. Several studies have also demonstrated that p210 protein fusion region-derived peptides bind to different MHC class-I molecules (A11 ,A0201 ,A3 and B8) and induce CML-specific cytotoxic T lymphocytes (CTL), which can kill CML cells *in vitro* [20, 22–26]. In addition, *in vivo*, bcr-abl fusion protein-specific CTLs were detected in PBMCs from CML patients using bcr-abl peptide/MHC tetramer technology [23]. Moreover, some leukemic-associated antigens such as WT1 and proteinase 3 (PR3), could also induce to yield CML-specific CTLs [15]. These CML-specific CTLs may consist of the same or different αβ^+^T cell clones. The specific method for identifying antigen-specific TCRs from CML-specific CTLs is peptide/MHC tetramer technology; however, few specific TCRs have been identified from CML using peptide/MHC tetramer technology. Oligoclonally expanded T cells may dominate an immune response induced by malignant cells, thus exhibiting a limited set of TCRα or β chains. Therefore, analysis of TCR usage in patients with leukemia is an excellent method for evaluating leukemic-specific immune responses [27]. RT-PCR-Genescan is one of the sensitive methods used to analyze TCR usage and detect oligoclonal T cell expansion [28], and it is widely used to detect antigen-specific TCR Vβ and Vα genes in infectious diseases, tumors, and autoimmune diseases [29, 30]. We previously observed the oligoclonal TCR Vβ21 gene at a high frequency (26.4%) in CML using RT-PCR-Genescan technology, and *in vitro* oligoclonal Vβ21 T cells from cord blood could be induced by K562 cells, CML cells,as well as a BCR-ABL fusion peptide. Furthermore, the emergence of oligoclonal Vβ21 T cells was investigated in relapse CML patients post allo-HSCT after DLI, and clonality was retained until complete remission [14, 31, 32]. This evidence suggests that clonally expanded Vβ21 T cells may have an anti-CML function.

Because TCRβ is just one chain in the TCR α/β complex, TCRβ clonal proliferation will theoretically accompany TCRα clonal proliferation. Thus in this study, the oligoclonally expanded TCR α13 and α18 genes were isolated from patients with CML using RT-PCR-Genescan technology, and these genes, which pair with Vβ21, were cloned into the pIRES eukaryotic expression plasmid (TCR Vα13-IRES-Vβ21 and TCR Vα18-IRES-Vβ21). The two recombinant plasmids were transfected into CD3^+^ T cells from HLA-A11^+^ healthy donors to determine their specific cytotoxicity *in vivo* cytotoxicity assays. Using the TCR gene cloned from a CML patient carrying HLA-A11, we artificially established an HLA-A11^+^ K562 cell line expressing the same HLA-A11 molecules as that in the CML patient as well as the target cells HLA-A11^+^K293 and pEGFP-N3^+^K562, which expresses GFP as a negative control. Both the TCR Vα13/Vβ21 and TCR Vα18/Vβ21 gene-modified T cells demonstrated specific cytotoxicity for HLA-A11^+^K562 cells. However, the specific cytotoxicity for the TCR Vα13/Vβ21 gene-modified T cells was superior to that of the TCR Vα18/Vβ21 gene-modified T cells. Both TCR gene-modified T cells demonstrated non-specific cytotoxicity for HLA-A11^+^K293 and pEGFP-N3^+^K562 cells; thus, specific anti-CML activity could be verified in this study. In addition, the granular enzyme and perforin gene transcription level and INF-γ level of the TCR Vα13/Vβ21 gene-modified T cells, which were co-cultured with HLA-A11^+^K562 cells, corresponded to the cytotoxic capacity. These results further demonstrated that TCR Vα13/Vβ21 cells have the ability of specifically recognizing CML cells, however, which tumor antigen peptide recognized by TCR Vα13/Vβ21 remains unclear. Tumor antigens can be broadly categorized into two types – those that are well defined and others that are undefined [33]. *In vitro* defined tumor antigen could be used to induce normal T cells to generate antigen specific T cell clone, then tumor antigen specific TCRs were indentified from these clone. This approach of using defined tumor antigens has been most widely explored in trials of individual antigens [12, 34, 35]. An advantage of using defined antigens for screening of candidate TCRs is easy to monitor specific immune response. However, this approach have been limited due to number of known tumor antigens for use. Alternative method to identify anti-tumor TCRs was to generate anti-tumor T cell clone expanded from tumor infiltrating lymphocyte (TIL) or induced by intact tumor cells or cell lysate [36]. The disadvantage is that defined antigens recognized by the indentified TCRs were unclear. Similar to the later method, in this study, we directly characterized the clonally expanded TCR clones from CML patients and confirmed their cytotoxicity by gene transfer techniques, regardless the defined tumor antigen, this may be the feasible approach for constitution of leukemia-specific T cell clone. Even we were unable to define the specific antigen of TCR Vα13/Vβ21 from oligoclonal T cells of patient with CML, TCR Vα13/Vβ21 is still a potential candidate for TCR gene therapy for CML based on their specific cytotoxicity against HLA-A11^+^K562 cells.

TCR gene therapy are based on changing T cell specificity through the expression of αβ TCRs for mediating the specific antigen recognition process. The procedure include identification of TCR α and β chains specific for tumor or leukemia associated antigens and construction of recombinant vectors and transfection of T cells generates tumor antigen specific T cells [37]. In the study, the identification of CML associated antigen specific TCR Vα13/Vβ21 was the beginning of TCR gene therapy for CML. For clinical applications in the future, the efficient and safety transfection system, TCR misspairing and TCR affinity and avidity must be further solved. Transfer of tumor antigen-specific TCRs to T cells requires a delivery system that yields high-level transfection efficiency and stable transgene expression. Retroviral and lentiviral systems are often using in most clinical studies. These systems are time-consuming and mediate genotoxic side effects. Alternative, more cost-effective and safety, plasmid-based gene transfer systems could offer a way to accelerated screening of a more variety of high affinity tumor antigen specific TCRs [38]. In this study, the selective bicistronic vector pIRES was relative suitable for rapid identifying the antigen specific TCR, which construct simply and fastly and inexpensive, but this plasmid system could not yields stable transgene expression, which limited its clinical application. Future trials, we will select plasmid-based gene transfer systems based on transposable elements such as sleeping beauty (SB) transposon to overcome the problem [39]. TCR mispairing could significantly weaken the functional avidity of the TCR gene modified T cells, because it would reduce the ability of the cells to recognize the desired target peptide and, meanwhile, it can represent a potential risk of autoimmunity [40]. Based on the identified TCR Vα13/Vβ21, we can employed numerous strategies to minimise the risk of mispairing, including murinization of human TCR-constant regions [41], cysteine modification of TCR chains, modifications of TCR constant chains [42, 43], alternation of conventional αβ TCRs using chimeric antigen receptors [44, 45] and introduction of TCR αβ chains into alternative effector cells [46, 47]. In addition, cytotoxicity assays for different CML cell lines with a large cohort should be performed to confirm the anti-CML cytotoxicity.

More recently, advance immunotherapy for leukemia focused on CAR-T cells such as CD19-CAR-T for CD19^+^ lymphoid leukemia, and bispecific antibody such as a bispecific T cell engager antibody (BiTE, Blinatumomab), against CD19/CD3 for refractory acute lymphoid leukemia, however, the application is mostly limited in B cell lymphoid leukemia which express CD19, CD20 or CD22 etc [48–50]. Antibody-based therapies, including CAR T cells and bispecific antibody, need recognise surface proteins. However, cell surface proteins as potential tumor targets are few in number, so the application of CAR T cells and bispecific antibody are limited in leukemias which carry specific biomarker in cell surface [51]. So far, none surface specific antigens were found on the cell surface of CML cell, immunotherapy based on TCR-modified CTL may be one of the best approachs. TCRs access both cell surface and intracellular proteins, so theoretically tumor antigen specific TCR gene therapy can be used to treat all tumors.

In summary, to our knowledge, this is the first report identifying CML-associated, antigen-specific TCR Vα13/Vβ21. This report provides the preliminary demonstration that TCR Vα13/Vβ21 gene-modified T cells have acquired specific anti-CML cytotoxicity. This study provides substantial new data for better understanding TCR-mediated gene therapy in CML.

## MATERIALS AND METHODS

### Samples from a CML patient and healthy donors

PBMCs were collected from a CML patient (HLA-A*11) after signed informed consent, and PBMCs from three healthy donors served as control. All procedures were conducted according to the guidelines of the Medical Ethics Committees of the Health Bureau of the Guangdong Province of China, and ethical approval was obtained from the Ethics Committee of Medical School of Jinan University.

### Cell lines

K562 cells, Jurkat T cells and HEK-293 cells were cultured in RPMI 1640 supplemented with 10% heat-inactivated fetal bovine serum (FBS), 20 mM HEPES, 0.5 mM sodium pyruvate, 100 U/ml penicillin, 100 μg/mL streptomycin, and Glutamax (Invitrogen, Carlsbad, CA).

### Construction of TCR Vα13-IRES-Vβ21 and Vα18-IRES-Vβ21 recombinant plasmids

The CML associated-TCR Vα13, Vα18 and TCR Vβ21 chain genes were isolated from a CML patient [14]. To clone the TCR genes, total RNA was extracted from the PBMCs of the CML patient, and it was reverse transcribed into cDNA. The cDNA was amplified by PCR using forward (VA13-F, VA18-F and VB21-F) and reverse (CA-R, CB-R) primers (Table 1). To construct the TCR Vα13-IRES-Vβ21 and Vα18-IRES-Vβ21 recombinant plasmids, the isolated TCR Vα13 and Vα18 genes were cloned into MCS A of the pIRES vector using the Nhe1 and EcoR1 restriction sites, and the isolated Vβ21 gene was cloned into MCS B of the TCR Vα13-IRES and TCR Vα18-IRES vectors using Xbal 1 and Sal1 restriction sites. The recombinant plasmids were then transfected into HEK 293 and Jurkat T cells using lipofectin and Nucleofector™ technology (Amaxa, Cologne, Germany), and the TCR α13, α18 and β21 proteins were detected using a TCR α and β monoclonal antibody (mAb) with laser confocal microscopy (LCM; 510 META DuoScan, Carl Zeiss, Germany) and flow cytometry (FCM) 24 hours after transfection.

**Table 1:** The sequence of primers for PCR

Primers	Sequence	Function
VA13-F	5′-TCGGCTAGCGGAGCAAGAAGGC AAAGCATC-3′	Sense primer for TCR α13 genes in the 5′ position of IRES containing ***Nhe*** ***I*** restriction enzyme sites
VA18-F	5′-TCGGCTAGCTGAGCAGGAAACA TGGAGAAGAAT-3′	Sense primer for TCR α18 genes in the 5′ position of IRES containing ***Nhe*** ***I*** restriction enzyme sites
CA-R	5′-ATTGAATTCGCGAGGGAGCACA GGCTGTCTTAC-3′	Antisense primer for TCR α13 and α18 genes in the 3′ position of IRES containing ***EcoR*** ***I*** restriction enzyme sites
VB21-F	5′-TAAGCTAGCCTCCCATCCTTCCC TGACCCT-3′	Sense primer for TCR β21 genes in the 5′ position of IRES containing ***Nhe*** ***I*** restriction enzyme sites
CB-R	5′-GCCGAATTCTCAGCCTCTGGAAT CCTTTCTCTTGAC-3′	Antisense primer for TCR β21 genes in the 3′ position of IRES containing ***EcoR*** ***I*** restriction enzyme sites
grmb-f	5′- AGATGCAACCAATCCTGCTT-3′	Sense primer for granzyme B
grmb-r	5′-CATGTCCCCCGATGATCT-3′	Antisense primer for granzyme B
perf-f	5′- CCGCTTCTCTATACGGGATTC-3′	Sense primer for perforin
perf-r	5′- GCAGCAGCAGGAGAAGGAT-3′	Antisense primer for perforin
gapdh-f	5′-AGCCACATCGCTCAGACAC-3′	Sense primer for GAPDH
gapdh-r	5′-GCCCAATACGACCAAATCC-3′	Antisense primer for GAPDH

### Western blot analysis

HEK 293 cells were harvested 24 hours after transfection, mixed with RIPA lysis buffer (1 × PBS, 1% Nonidet P-40, 0.5% sodium deoxycholate, 0.1% sodium dodecyl sulfate [SDS], 10 mmol/L phenylmethylsulfonyl fluoride, 1 μg/mL aprotinin, and 100 mmol/L sodium orthovanadate), and incubated on ice for 30 min to isolate total proteins. Proteins (100 μg) were separated by 7.5% SDS-PAGE and transferred to nitrocellulose membranes (Invitrogen, USA) using a damp-dry transfer device (Bio-rad, USA). After blocking for 1 hours in 5% defatted milk powder in PBS, the membranes were washed and probed with a mouse anti-human TCR β monoclonal antibody and a rabbit anti-human TCRα polyclonal antibody (Santa Cruz, USA). Similar studies were performed with 1:500 mouse-anti-human β-actin (BOSTER, Wuhan, China). The antibodies were detected using 1:10,000 horseradish peroxidase-conjugated, goat-anti-rabbit IgG and donkey-anti-mouse IgG (Jackson ImmunoResearch, USA). A Western blotting luminol reagent (Tiangen, Beijing, China) was used to visualize bands corresponding to each antibody.

### Human CD3^+^T cell isolation and culture

Peripheral blood mononuclear cells (PBMCs) obtained from three healthy donors (HLA-A*11, DP restricted) were isolated from heparinized venous blood by Ficoll-Paque gradient centrifugation. Cells were collected, washed twice in Hank's balanced salt solution, and resuspended at a final concentration of 2 × 10^6^ cells/mL in T cell medium OpTmizer^™^ SFM (Invitrogen, Grand Island, NY) supplemented with 2% heat-inactivated fetal calf serum (FCS; HyClone, Logan, UT), 100 U/mL penicillin, 100 μg/mL streptomycin, 2 mM L-glutamine, and 50 μM 2-mercaptoethanol. CD3^+^T cells were purified from freshly isolated PBMCs using CD3^+^ microbeads (Miltenyi Biotec, Bergisch Gladbach, Germany) according to the manufacturer's protocol. The purity of the collected CD3^+^T cells was assessed by flow cytometry. Greater than 95% of the CD3^+^T cells were collected using this technique. Initial stimulation was performed at a concentration of 2 × 10^6^ cells per well in 1 mL T cell medium OpTmizer^™^ SFM (containing 200 IU/mL IL-2, 1 μg/mL OKT3 and 2 μg/mL CD28 monoclonal antibody) for 24 h in non-tissue culture 12-well plates. Cells were washed once with medium on the following day and then added to fresh complete RPMI 1640 medium supplemented with 200 IU/ml IL-2.

### Artificial killing of target cell lines

To generate artificial killing of target cells, the K562 cell line was modified to express human HLA-A*11 with an HLA-A*11-2A-eGFP vector using transduction. After transduction, K562 cells were selected by drug resistance (G418) and the limited dilution method to obtain an HLA-A*11^+^ K562 cell clone [16]. This clone was then expanded, and the expression of the transgenic molecules was monitored by fluorescence-activated cell sorting (FACS) over time. In addition, the pEGFP-N3^+^ K562 and HLA-A11^+^K293 cell clones were constructed as negative control cell lines.

### Transduction of TCR genes in T cells

Human CD3^+^T cells were transfected using Nucleofector™ technology (Amaxa, Cologne, Germany). Briefly, cells (1 × 10^7^) were resuspended in 0.1 mL supplemented Nucleofector solution at room temperature from the human T cell Nucleofector ™ kit. Each plasmid (5 μg; including the TCR Vα13-IRES-Vβ21 and TCR Vα18-IRES-Vβ21 recombinant plasmids and the negative control TCR empty vector) was mixed with 0.1 mL cell suspension, transferred to a 2.0 mm electroporation cuvette, and nucleofected using an Amaxa Nucleofector II apparatus according to the manufacturer's guidelines. Storage of the cell suspension in human T cell Nucleofector solution for longer than 20 min was avoided as this reduces cell viability and gene transfer efficiency. The cells were transfected using the U-014 program. The transfected T cells were immediately transferred to pre-warmed T cell medium OpTmizer^TM^ SFM and cultured in 12-well plates in a humidified incubator at 37°C and 5% CO_2_. The culture medium was changed at 4–6 and 18 hours after transfection to T cell medium containing 200 IU/mL IL-2 and 2% FCS. The monoclonal antibodies 1 μg/mL OKT3 and 2 μg/mL CD28 were later added, and the cells were cultured for 48 hours. T cells were stained with CD3-PE and Vβ21.3-PE (Beckman Coulter, California, USA) to monitor TCR expression. Flow cytometry was performed using a FACS Canto flow cytometer (BD Biosciences).

### Calcein-acetyoxymethyl cytotoxicity assay

Calcein-AM was purchased from Molecular Probes (Eugene, OR) as a 1 mg/ml solution in dry dimethyl sulfoxide. Target cells (pEGFP-N3^+^ K562 cell, HLA-A11^+^ K562 cell, and HLA-A11^+^K293 cell) were resuspended in complete medium at a final concentration of 10^3^/μL and incubated with 15 μM calcein-AM for 30 min at 37°C with occasional shaking. After two washes in complete medium, cells were adjusted to 10^2^ /μL in U bottom 96-well microtiter plates (Nunc, USA) with an E:T ratio of 40:1 in triplicate with at least six replicate wells for spontaneous (only target cells in complete medium) and maximum release (target cells in the medium alone plus 2% Triton X-100). The numbers of TCR gene-transferred T cells (effector cells) and labeled target tumor cells were seeded as follows: each well contained 2 × 10^5^ lymphocytes in 100 μL complete medium and 5 × 10^3^ target cells/50 μL of complete medium. After incubation at 37°C in 5% CO_2_ for 4 h, 75 μL of each supernatant was harvested and transferred into new plates. Samples were measured using an ELX 800 absorbance reader (Bio-TEK, USA) (excitation filter: 485 ± 9 nm; band-pass filter: 530 ± 9 nm). Data were expressed as arbitrary fluorescent units (AFU). Specific lysis was calculated according to the following formula: [(experimental release-target spontaneous release-effector spontaneous release)/(target maximum release-target spontaneous release)] × 100%.

### Quantitative real-time reverse transcription (RT)-PCR analysis

After the calcein-AM cytotoxicity assay, total RNA from mixed cells was extracted using the RNeasy Mini kit (Qiagen, Valencia, CA) following the manufacturer's instructions. Granzyme B and perforin mRNA were measured by real-time quantitative reverse transcription polymerase chain reaction (qRT-PCR) analysis using SYBR^®^ Green I with the Real Master Mix kit (Tiangen, Beijing, China). Reactions were run in triplicate and repeated in three independent experiments using the CFX real-time PCR system (Bio-Rad, USA) with cDNA template in a 25 μL reaction under the following conditions: 95°C for 2 min followed by 45 cycles of 95°C for 15 s, 65°C for 15 s and 72°C for 40 s. The primers used for real-time PCR are listed in Table 1 as described by Nagai K *et al.* [52].

### ELIspot assay

The IFN-γ ELIspot assay was performed as described by Paret C *et al.* [35]. Briefly, the TCR gene-transferred T cells were plated in triplicate and serially diluted at 2 × 10^4^ cells/well, and 8 × 10^4^ target cells that were treated with Mitomycin-C were added. In all experiments, TCR gene-transferred T cells were also incubated with free medium as a negative control. Plates were incubated in 5% CO_2_ at 37°C for 24 hours. The medium and cells were removed and 200 μL deionized water was added and incubated on ice for 10 min. After washing ten times with PBS containing 0.05% Tween-20, 100 μL biotinylated anti-IFN-γ antibody was added to each well, and plates were incubated at 37°C for 1 hour. The plates were washed again and incubated with HRP-labeled streptavidin at 37°C for 1 h. After washing the plates again, 100 μl of AEC solution was added to each well, and the plates were incubated at room temperature for 30 min. The color reaction was stopped by washing the plates with deionized water. The spots were counted with a body vision microscope; each spot represented an IFN-γ-secreting cell, and the average of the three wells was calculated as the detection value. The number of specific CTLs was determined as spots per 20,000 effector cells.

### Statistical analysis

The Mann-Whitney test for two independent samples was used to determine differences between groups using SPSS 11.5 statistical software. Differences with a *P* < 0.05 were considered statistically significant.
